# The effects of war on patients with Parkinson's disease

**DOI:** 10.1016/j.prdoa.2026.100491

**Published:** 2026-07-27

**Authors:** Mehri Salari, Masoud Etemadifar, Kamran Rezaei, Fatemeh Hojjatipour

**Affiliations:** a Men's health and reproductive health research center, Shahid Beheshti University of Medical Sciences, Tehran, Iran; b Neurosurgery Department, Isfahan University of Medical Sciences, Isfahan, Iran; c School of Medicine, Shahid Beheshti University of Medical Sciences, Tehran. Iran

In this study, we aim to evaluate whether patients perceived more stress than healthy controls after the conflict between Iran and Israel and what symptoms were correlated with this stress, measured by the Impact of Event Scale-Revised (IES-R). However, PD patients exhibited significantly lower stress levels than healthy controls.

Parkinson's Disease (PD) is the second most common neurodegenerative movement disorder. It is stated that chronic stress can be a potential trigger factor for PD occurrence [1]. Moreover, people with PD (PwPD) are very vulnerable to the negative effects of psychological distress [2]. One of these stressful phenomena that can cause excessive challenges for patients is war.

In this observational cross-sectional study, an online survey was conducted to assess post-traumatic stress disorder using the Persian-language version of the IES-R, which has been validated in Iran with good psychometric properties [3] in PD patients. This study was done in the context of a recent armed conflict in Iran that occurred from June 2025 13th to 25th. Data were collected from a week after the war and continued for more than two months. The population of the study was adults (age ≥ 18 years) with a confirmed diagnosis of PD by the Movement Disorder Society Criteria by a movement disorder specialist. They should also have been in Iran during the time of the conflict. Written informed consent was obtained. We removed any PD patients who had been diagnosed with significant mental or stress-related disorders before the war. A comparison group of healthy controls (HC) who lived in Iran during the conflict was chosen from volunteers or hospital staff and was age- and sex-matched to the patients. The questions were based on the feelings of subjects during and after the war for about a week.

Our self-report questionnaire included demographic information (age, sex, and residence city, duration of PD), disease activity, (dyskinesia, bradykinesia, tremor, and balance), changes in their drug dosage, whether they needed anxiety medications, whether they needed to see a doctor, whether the doctor was available, whether their drug access was interrupted, changes in drug usage, and the IES-R questionnaire. Responses were collected on a yes/no basis. We also inquired about general exposure to war stressors, categorized as none, moderate, or high, to provide descriptive context. To minimize missing data, most critical questions were mandatory. These variables were all measured once after the war.

We used the Shapiro-Wilk test to verify normality in continuous variables. Using independent *t*-tests for continuous parameters and chi-square testing for categorical features, we compared the baseline demographics of the PwPD and HC groups. Additionally, we compared their distress after the war using an ANCOVA test to adjust for the sex and age of the patients and controls.

To evaluate the IES-R score and the likelihood of symptom exacerbation, we also conducted a logistic regression analysis, controlling for variables such as age, sex, and length of illness. Also, we used an ordinal logistic regression where we divided the IES-R score into three groups: 0, no PTSD is present; 1, a possibility of PTSD; and 2, indicates a presence of PTSD. For all tests, a two-tailed *p*-value < .05 was deemed statistically significant.

14.8% reported that their dosage had changed because of changes in their symptoms, 38.3% reported that their tremor had gotten worse, 37% reported that their bradykinesia had gotten worse, 14.8% reported that they had fallen more than before, 27.2% reported that their dyskinesia had gotten worse, and 29.6% reported increased dosage or adding medication to treat their anxiety during this period. 30.9% of patients said they needed to make an appointment with a doctor (17.3% said the doctor wasn't available), 27.2% said they had trouble getting their meds, 32% of DBS patients said they needed adjustments, and none of the Apomorphine pump patients needed adjustments.

Analysis of covariance showed those with the disease had lower total scores than controls (*p* < .001). There was also a significant main effect of gender (*p* = .024), with women scoring higher than men. In ordinal regression analysis, correlations between increased stress intensity and symptom exacerbation were seen. These results show that, regardless of clinical and demographic variables, stress-related deterioration is most noticeable in tremor, bradykinesia, and anxiety. All of the demographic variables, linear regression analysis results, and results from ordinal regression analysis are summarized in Table 1.

**Table 1:** Demographic Characteristics and Regression Analyses of IES-R Outcomes.

Variable / Predictor	Category	Patients (*n* = 81)	Controls (*n* = 74)	B	SE	95% CI	β / OR	p-value
**Demographic characteristics of patients and healthy controls**								
Number		81	74					
Gender (M/F)		51/30	26/48					
Age, mean ± SD		59.93 ± 11.74	42.22 ± 15.42					
IES-R, mean ± SD		18.53 ± 18.7	31.55 ± 17.53					
IES-R categorized, %	0	71.6	39.2					
	1	6.2	16.2					
	2	22.2	44.6					

**Linear regression analysis between variables and the IES-R score**								
Tremor exacerbation	1 vs. 0			12.810	4.191	4.49–21.13	0.332	0.003
Bradykinesia exacerbation	1 vs. 0			12.643	4.151	4.40–20.89	0.329	0.003
Falling exacerbation	1 vs. 0			12.403	5.669	1.15–23.66	0.237	0.032
Anxiety exacerbation	1 vs. 0			9.992	4.399	1.24–18.74	0.246	0.026
Increased doctor visits	1 vs. 0			8.603	4.491	−0.34–17.55	0.214	0.059
Dyskinesia exacerbation	1 vs. 0			4.623	4.723	−4.79–14.04	0.111	0.331
Reduced medication availability	1 vs. 0			3.633	10.130	−16.57–23.84	0.082	0.724
Increased medication use	1 vs. 0			−0.208	4.613	−9.48–9.06	−0.005	0.964
DBS status	1 vs. 0			12.727	11.345	−9.82–35.27	0.129	0.266
Apomorphine users	1 vs. 0			19.990	10.005	0.09–39.89	0.233	0.049

**Ordinal regression analysis between variables and the IES-R category**								
Tremor	No change vs. Yes					0.059–0.508	0.173	0.001
Bradykinesia	No change vs. Yes					0.080–0.651	0.232	0.006
Anxiety	No change vs. Yes					0.111–0.897	0.316	0.030
Increased doctor visits	No change vs. Yes					0.141–1.164	0.406	0.093
Falling	No change vs. Yes					0.082–1.080	0.296	0.065
Dyskinesia	No change vs. Yes					0.132–1.138	0.387	0.085
DBS status	No change vs. Yes					0.032–3.999	0.356	0.403
Medication availability	No change vs. Yes					0.049–2.367	0.339	0.276
Increased medication use	No change vs. Yes					0.552–6.347	1.872	0.315
Apomorphine users	No change vs. Yes					0.026–2.287	0.243	0.216

Notes

IES-R: Impact of Event Scale-Revised. SD: Standard Deviation. M: Male. F: Female.

Linear regression: B = unstandardized regression coefficient (mean difference in IES-R score); SE = standard error; 95% CI = confidence interval for B; β = standardized coefficient; *p*-value from two-tailed test. All models adjusted for age, gender (1 = male, 0 = female), and illness duration. The reference group for each binary predictor is the absence of the condition/change.

Ordinal regression: OR = odds ratio; CI = confidence interval; p = *p*-value.

The β / OR column reports the standardized coefficient (β) for the linear regression section and the odds ratio (OR) for the ordinal regression section; these are distinct statistics and are not directly comparable across sections.

According to our results, falls and anxiety were much more common in this period. However, the patients experienced less distress than HCs caused by war, a traumatic event, according to the IES-R.

The loss of social surroundings, disruption of life direction, less control over one's life, anxiety-inducing news updates, and lack of access to resources that meet basic and emotional requirements are all factors that lead to psycho-emotional vulnerability as a result of migration in war. However, chronic patients suffer from more issues, such as shortage of medical supplies, rehabilitation, and critical healthcare services. These medical, physical, and neurological changes can exacerbate mental illnesses like PTSD, anxiety, depression, and drug and alcohol addiction.

In contrast to our result, Salari et al. [4] stated that during the pandemic, PwPD had higher levels of stress, sadness, anxiety, and physical activity than controls. These findings may be explained by the fact that patients typically experience anxiety far more than HC at baseline, and changes in stress and anxiety following an event are not measured. Also, we used a different measurement tool, which may affect the results.

Moreover, Nistico et al. [5] revealed that there was no difference in IES-R between PwPD and HC during the pandemic, and PD patients' general health remained constant or even improved.

These different results might be due to different factors such as acute/chronic phase of war effects, subjective or objective measurements, and the fact that PwPD in war were able to stay with their families and had many more gatherings with their loved ones in contrast to the COVID-19 lockdown. On the other hand, PwPD with difficulties with social interactions or those with comorbid anxiety might have benefited from moving to cities other than the capital, since they did not have to face external factors [5].

In this study, we tried to address challenges PwPD faced after the 12-day war in Iran. This is significantly important because it will inform patients, physicians, and governments to address the challenges these patients experience.

## Ethical Compliance Statement

1. Shahid Beheshti University of Medical Sciences' ethics committee approved this study.
  2. Written informed patient consent was taken at the beginning of the questionnaire.
  3. We confirm that we have read the Journal's position on issues involved in ethical publication and affirm that this work is consistent with those guidelines.

## CRediT authorship contribution statement

**Mehri Salari:** Writing – review & editing, Supervision, Methodology, Investigation, Conceptualization. **Masoud Etemadifar:** Writing – review & editing, Supervision, Project administration, Methodology, Investigation, Conceptualization. **Kamran Rezaei:** Writing – original draft, Conceptualization. **Fatemeh Hojjatipour:** Writing – review & editing, Writing – original draft, Validation, Project administration, Methodology, Investigation, Formal analysis, Conceptualization.

## Funding Sources

No specific funding was received for this work, and the authors declare that there are no conflicts of interest relevant to this work.

## Declaration of competing interest

The authors declare that they have no known competing financial interests or personal relationships that could have appeared to influence the work reported in this paper.
